# TSC-associated neuropsychiatric disorders (TAND): findings from the TOSCA natural history study

**DOI:** 10.1186/s13023-018-0901-8

**Published:** 2018-09-10

**Authors:** Petrus J. de Vries, Elena Belousova, Mirjana P. Benedik, Tom Carter, Vincent Cottin, Paolo Curatolo, Maria Dahlin, Lisa D’Amato, Guillaume B. d’Augères, José C. Ferreira, Martha Feucht, Carla Fladrowski, Christoph Hertzberg, Sergiusz Jozwiak, J. Chris Kingswood, John A. Lawson, Alfons Macaya, Ruben Marques, Rima Nabbout, Finbar O’Callaghan, Jiong Qin, Valentin Sander, Matthias Sauter, Seema Shah, Yukitoshi Takahashi, Renaud Touraine, Sotiris Youroukos, Bernard Zonnenberg, Anna C. Jansen, on behalf of TOSCA Consortium and TOSCA Investigators, Nobuo Shinohara, Shigeo Horie, Masaya Kubota, Jun Tohyama, Katsumi Imai, Mari Kaneda, Hideo Kaneko, Yasushi Uchida, Tomoko Kirino, Shoichi Endo, Yoshikazu Inoue, Katsuhisa Uruno, Ayse Serdaroglu, Zuhal Yapici, Banu Anlar, Sakir Altunbasak, Olga Lvova, Oleg Valeryevich Belyaev, Oleg Agranovich, Elena Vladislavovna Levitina, Yulia Vladimirovna Maksimova, Antonina Karas, Yuwu Jiang, Liping Zou, Kaifeng Xu, Yushi Zhang, Guoming Luan, Yuqin Zhang, Yi Wang, Meiling Jin, Dingwei Ye, Weiping Liao, Liemin Zhou, Jie Liu, Jianxiang Liao, Bo YAN, Yanchun Deng, Li Jiang, Zhisheng Liu, Shaoping Huang, Hua Li, Kijoong Kim, Pei-Lung Chen, Hsiu-Fen Lee, Jeng-Dau Tsai, Ching-Shiang Chi, Chao-Ching Huang, Kate Riney, Deborah Yates, Patrick Kwan, Surachai Likasitwattanakul, Charcrin Nabangchang, Lunliya Thampratankul Krisnachai Chomtho, Kamornwan Katanyuwong, Somjit Sriudomkajorn, Jo Wilmshurst, Reeval Segel, Tal Gilboa, Michal Tzadok, Aviva Fattal-Valevski, Panagiotis Papathanasopoulos, Antigone Syrigou Papavasiliou, Stylianos Giannakodimos, Stylianos Gatzonis, Evangelos Pavlou, Meropi Tzoufi, A. M. H. Vergeer, Marc Dhooghe, Hélène Verhelst, Filip Roelens, Marie Cecile Nassogne, Pierre Defresne, Liesbeth De Waele, Patricia Leroy, Nathalie Demonceau, Benjamin Legros, Patrick Van Bogaert, Berten Ceulemans, Lina Dom, Pierre Castelnau, Anne De Saint Martin, Audrey Riquet, Mathieu Milh, Claude Cances, Jean-Michel Pedespan, Dorothee Ville, Agathe Roubertie, Stéphane Auvin, Patrick Berquin, Christian Richelme, Catherine Allaire, Sophie Gueden, Sylvie Nguyen The Tich, Bertrand Godet, Maria Luz Ruiz Falco Rojas, Jaume Campistol Planas, Antonio Martinez Bermejo, Patricia Smeyers Dura, Susana Roldan Aparicio, Maria Jesus Martinez Gonzalez, Javier Lopez Pison, Manuel Oscar Blanco Barca, Eduardo Lopez Laso, Olga Alonso Luengo, Francisco Javier Aguirre Rodriguez, Ignacio Malaga Dieguez, Ana Camacho Salas, Itxaso Marti Carrera, Eduardo Martinez Salcedo, Maria Eugenia Yoldi Petri, Ramon Cancho Candela, Ines da Conceicao Carrilho, Jose Pedro Vieira, José Paulo da Silva Oliveira Monteiro, Miguel Jorge Santos de Oliveira Ferreira Leao, Catarina Sofia Marceano Ribeiro Luis, Carla Pires Mendonca, Milda Endziniene, Jurgis Strautmanis, Inga Talvik, Maria Paola Canevini, Antonio Gambardella, Dario Pruna, Salvatore Buono, Elena Fontana, Bernardo Dalla Bernardina, Carmen Burloiu, Iuliu Stefan Bacos Cosma, Mihaela Adela Vintan, Laura Popescu, Karel Zitterbart, Jaroslava Payerova, Ladislav Bratsky, Zuzana Zilinska, Ursula Gruber-Sedlmayr, Matthias Baumann, Edda Haberlandt, Kevin Rostasy, Ekaterina Pataraia, Frances Elmslie, Clare Ann Johnston, Pamela Crawford, Peter Uldall, Paul Uvebrant, Olof Rask, Marit Bjoernvold, Eylert Brodtkorb, Andreas Sloerdahl, Ragnar Solhoff, Martine Sofie Gilje Jaatun, Marek Mandera, Elzbieta Janina Radzikowska, Mariusz Wysocki, Michael Fischereder, Gerhard Kurlemann, Bernd Wilken, Adelheid Wiemer-Kruel, Klemens Budde, Klaus Marquard, Markus Knuf, Andreas Hahn, Hans Hartmann, Andreas Merkenschlager, Regina Trollmann

**Affiliations:** 1 Division of Child and Adolescent Psychiatry, University of Cape Town, 46 Sawkins Road, Rondebosch, Cape Town, 7700 South Africa; 2 Research and Clinical Institute of Pediatrics, Pirogov Russian National Research Medical University, Moscow, Russian Federation; 3 SPS Pediatrična Klinika, Ljubljana, Slovenia; 4 TSA Tuberous Sclerosis Association, Nottingham, UK; 5 Hôpital Louis Pradel, Claude Bernard University Lyon 1, Lyon, France; 6 Tor Vergata University Hospital, Rome, Italy; 7 Karolinska University Hospital, Stockholm, Sweden; 8 Novartis Farma S.p.A, Origgio, Italy; 9 Association Sclérose Tubéreuse de Bourneville, Gradignan, France; 10 Centro Hospitalar Lisboa Ocidental, Lisbon, Portugal; 11 Universitätsklinik für Kinder-und Jugendheilkunde, Vienna, Austria; 12 Associazione Sclerosi Tuberosa ONLUS, Milan, Italy; 13 European Tuberous Sclerosis Complex Association, In den Birken, Dattein, Germany; 14 Vivantes-Klinikum Neukölln, Berlin, Germany; 15 Department of Child Neurology, Warsaw Medical University, Warsaw, Poland; 16 Sussex Kidney Unit, Royal Sussex County Hospital, Brighton, UK; 17 The Tuberous Sclerosis Multidisciplinary Management Clinic, Sydney Children’s Hospital, Randwick, NSW Australia; 18 Hospital Universitari Vall d’Hebron, Barcelona, Spain; 19 Institute of Biomedicine, University of Leon, Leon, Spain; 20 Department of Pediatric Neurology, Necker Enfants Malades Hospital, Paris Descartes University, Paris, France; 21 Institute of Child Health, University College London, London, UK; 22 Department of Pediatrics, Peking University People’s Hospital (PKUPH), Beijing, China; 23 Tallinn Children Hospital, Tallinn, Estonia; 24 Klinikverbund Kempten-Oberallgäu gGmbH, Kempten, Germany; 25 Novartis Healthcare Pvt. Ltd, Hyderabad, India; 26 National Epilepsy Center, Shizuoka Institute of Epilepsy and Neurological Disorders, NHO, 886 Urushiyama, Aoi-ku, Shizuoka Japan; 27 Hôpital Nord, Saint Etienne, France; 28 “St. Sophia” Children’s Hospital, Athens, Greece; 29 University Medical Center, Utrecht, The Netherlands; 30 UZ Brussel Vrije Universiteit Brussel, Brussels, Belgium

**Keywords:** TSC-associated neuropsychiatric disorders, Tuberous sclerosis complex, TOSCA

## Abstract

**Background:**

Most evidence for TSC-associated neuropsychiatric disorders (TAND) to date have come from small studies and case reports, and very little is known about TAND in adults. We explored baseline TAND data from the large-scale international TOSCA natural history study to compare childhood and adult patterns, describe age-based patterns, and explore genotype-TAND correlations.

**Results:**

The study enrolled 2216 eligible participants with TSC from 170 sites across 31 countries at the data cut-off for the third interim analysis (data cut-off date: September 30, 2015). The most common behavioural problems (reported in > 10% of participants) were overactivity, sleep difficulties, impulsivity, anxiety, mood swings, severe aggression, depressed mood, self-injury, and obsessions. Psychiatric disorders included autism spectrum disorder (ASD, 21.1%), attention deficit hyperactivity disorder (ADHD, 19.1%), anxiety disorder (9.7%), and depressive disorder (6.1%). Intelligence quotient (IQ) scores were available for 885 participants. Of these, 44.4% had normal IQ, while mild, moderate, severe, and profound degrees of intellectual disability (ID) were observed in 28.1, 15.1, 9.3, and 3.1%, respectively. Academic difficulties were identified in 58.6% of participants, and neuropsychological deficits (performance <5th percentile) in 55.7%. Significantly higher rates of overactivity and impulsivity were observed in children and higher rates of anxiety, depressed mood, mood swings, obsessions, psychosis and hallucinations were observed in adults. Genotype-TAND correlations showed a higher frequency of self-injury, ASD, academic difficulties and neuropsychological deficits in *TSC2*. Those with no mutations identified (NMI) showed a mixed pattern of TAND manifestations. Children and those with *TSC2* had significantly higher rates of intellectual disability, suggesting that age and genotype comparisons should be interpreted with caution.

**Conclusions:**

These results emphasize the magnitude of TAND in TSC and the importance of evaluating for neuropsychiatric comorbidity in all children and adults with TSC, across *TSC1* and *TSC2* genotypes, as well as in those with no mutations identified. However, the high rates of unreported or missing TAND data in this study underline the fact that, even in expert centres, TAND remains underdiagnosed and potentially undertreated.

## Background

Tuberous sclerosis complex (TSC) is an autosomal dominant genetic disorder characterized by the formation of hamartomas in multiple organ systems with a wide diversity of symptoms and severity across individuals [1, 2]. The majority of individuals with TSC have central nervous system involvement with a wide range of structural manifestations, such as cortical tubers, subependymal nodules, and aberrations of gray and white matter as well as high rates of functional manifestations, such as epilepsy, intellectual disability (ID), and behavioural problems [3–5]. The neurological, neuropsychiatric, and renal manifestations represent the greatest burden of disease of all TSC-related manifestations [3].

TSC-associated neuropsychiatric disorders (TAND) is an umbrella term coined by the Neuropsychiatry Panel of the 2012 International Consensus Conference for TSC, and encompasses a range of neuropsychiatric manifestations across various levels of investigation [2, 3, 5]. These include the behavioural level (observed behaviours such as sleep problems or aggressive behaviours), the psychiatric level (DSM/ICD defined psychiatric disorders such as autism spectrum disorders [ASD] or attention deficit hyperactivity disorder [ADHD]), the intellectual level (intellectual ability as defined by intelligence quotient [IQ]-type tests), the academic level (learning disorders, e.g., reading or mathematics difficulties), and the psychosocial level (e.g., self-esteem, family difficulties) [3, 5, 6]. TAND represent a significant burden of disease and have a major impact on quality of life of individuals with TSC and their families, given their impact on education, employment, and social life of patients and their family [3, 5].

The rate and pattern of TAND vary greatly among patients with TSC [3, 5, 7–11]. Overall, about 90% of individuals with TSC exhibit TAND features to some extent during their lifetime, with ASD and ID reported in up to 50% of individuals [3, 5, 6]. Results show differential rates in those with and without ID [12]. However, even individuals with normal intellectual abilities are at risk of developing TAND manifestations, particularly in the academic, neuropsychological, and psychosocial domains [8]. Despite the high rates of TAND, a 2010 survey of members of the Tuberous Sclerosis Association in the United Kingdom indicated that only 20% of individuals with TSC had ever received any assessment or treatment for TAND, suggesting a large assessment and treatment gap [5, 6, 12, 13]. It is important to acknowledge that the majority of evidence for rates and patterns of TAND to date have come from relatively small studies and case reports. The largest published studies to date have included a few hundred participants [10, 12, 14]. Furthermore, very little TAND data are available about adults with TSC [15] or about the age-based pattern of TAND.

The underlying aetiology of TAND is likely to be combinatorial and multifactorial [16]. There is evidence that the genetic aberrations of TSC may be sufficient to cause TAND manifestations, [16, 17] with combinatorial and interactive contribution from functional and structural factors [16–22]. Individuals with *TSC2* mutations have been reported to have a greater likelihood of ID than those with *TSC1,* [23–26] but both *TSC1* and *TSC2* mutations have been associated with the full range of intellectual ability from high IQ to profound ID [25, 26]. Individuals with no mutation identified (NMI) after genetic testing have typically been described to have ID profiles between those with *TSC1* and *TSC2* [23–26]. These studies have also been based on relatively small samples and no study to date has examined other aspects of TAND in relation to genotype.

The largest natural history study of TSC to date – the TOSCA (TuberOus SClerosis registry to increase disease Awareness) study is a multi-centre, international disease registry designed with the aim of providing deeper insights into the manifestations of TSC and its management [27, 28]. In a previous publication outlining baseline findings from the TOSCA cohort of 2093 individuals, we presented topline findings of TAND features in the largest TSC cohort reported globally to date [28]. Results showed that ID was observed in 54% of the evaluated participants and suggested that psychiatric disorders were typically diagnosed late. We also identified significant non-reported or missing data, which suggested that even in expert TSC centres around the globe, TAND may be underdiagnosed and therefore under-treated [28].

Here, we performed a detailed exploration of the largest TAND dataset to date using TOSCA baseline data of all the patients enrolled at the cut-off of September 30, 2015, with the specific aim of comparing childhood and adult TAND profiles, describing the age-related pattern of TAND, and examining TAND in relation to genotype.

## Methods

A detailed description of the methods of the TOSCA registry has been provided previously [27]. In short, individuals of any age with a documented visit for TSC before 12 months of enrollment or newly diagnosed with TSC were included in the study between August 2012 and August 2014.

Information on participant demography, family history, genotype, vital signs, prenatal history, clinical features of TSC across all organ systems, comorbidities, and rare manifestations were collected, both retrospectively and prospectively at baseline and annually thereafter for up to 5 years. Given that this was a natural history study, participants were followed up based on clinical need, and no clinical, laboratory or formal TAND evaluations were mandated by the protocol. The terms and operationalization of TAND manifestations and ‘levels’ were defined as in the primary TAND publication [5].

The TOSCA registry was designed and conducted in accordance with the Guidelines for Good Clinical Practice and ethical principles outlined in the Declaration of Helsinki [29, 30]. Written informed consent, with prior endorsement by all local institutional review boards (human research ethics committees) was obtained from all participants, parents, or guardians prior to registry enrollment.

For the purposes of this manuscript, descriptive statistics were used to summarize TAND data. Frequency of TAND features were extracted and presented as percentage of individuals with available data (excluding non-reported or missing data). Intellectual ability was categorized as normal (IQ > 70), mild ID (IQ 51–70), moderate ID (IQ 36–50), severe ID (IQ 20–35), and profound ID (IQ < 20), according to DSM-5/ICD-10 [31, 32]. Psychiatric disorders were defined according to the DSM-5/ICD-10. Age-based patterns of TAND (children vs adults) and the association between TAND and genotype (*TSC1* vs *TSC2*) were analyzed using Chi-square test. The TOSCA study included data collection on all levels of TAND as outlined above and summarised in the primary TAND manuscript [5], apart from psychosocial characteristics, such as self-esteem, sibling, or family stressors, which were only included after the TOSCA database was set up.

## Results

A total of 2216 participants (1154 females and 1062 males) with TSC were enrolled into the TOSCA registry from 170 sites across 31 countries at the data cut-off for the third interim analysis (September 30, 2015). The median age of the TOSCA cohort was 13 years (range, < 1–71) with 1410 individuals (63.6%) aged ≤18 years.

### Overall TAND features

**Fig. 1:**
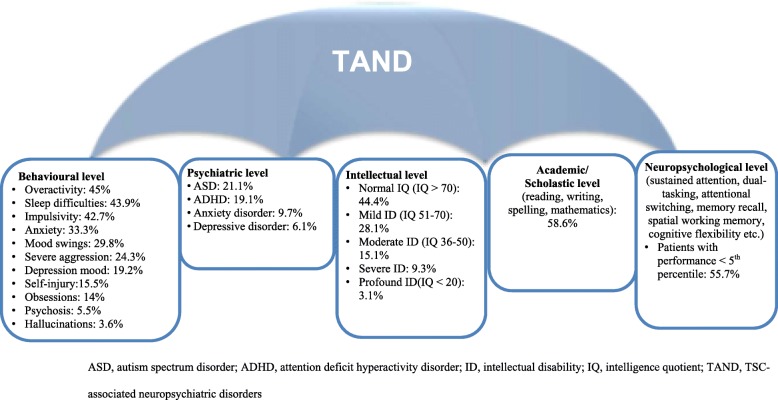
Summary of TAND Findings from the TOSCA Study (*N* = 2216)

Table 1 and Fig. 1 represent the frequencies of TAND features in the overall TOSCA cohort.

**Table 1:** TAND Features in the Overall TOSCA Cohort (*N* = 2216)

TAND Features	Individuals With Manifestation, n (%)^a^	Individuals With Available Data, n (%)	Individuals with Data Not Available*, n (%)
Behavioural level			
Overactivity	337 (45.0)	749 (33.8)	1467 (66.2)
Sleep difficulties	331 (43.9)	754 (34.0)	1462 (66.0)
Impulsivity	317 (42.7)	742 (33.5)	1474 (66.5)
Anxiety	240 (33.3)	720 (32.5)	1496 (67.5)
Mood swings	214 (29.8)	718 (32.4)	1498 (67.6)
Severe aggression	183 (24.3)	754 (34.0)	1462 (66.0)
Depression mood	139 (19.2)	724 (32.7)	1492 (67.3)
Self-injury	117 (15.5)	755 (34.1)	1461 (65.9)
Obsessions	100 (14.0)	714 (32.2)	1502 (67.8)
Psychosis	40 (5.5)	725 (32.7)	1491 (67.3)
Hallucinations	26 (3.6)	719 (32.5)	1497 (67.5)
Psychiatric level			
ASD	314 (21.1)	1486 (67.1)	730 (32.9)
ADHD	268 (19.1)	1404 (63.4)	812 (36.6)
Anxiety disorder	133 (9.7)	1365 (61.6)	851 (38.4)
Depressive disorder	84 (6.1)	1371 (61.9)	845 (38.1)
Intellectual level			
Normal	393 (44.4)	885 (39.9)	1331 (60.1)
Mild ID	249 (28.1)	885 (39.9)	1331 (60.1)
Moderate ID	134 (15.1)	885 (39.9)	1331 (60.1)
Severe ID	82 (9.3)	885 (39.9)	1331 (60.1)
Profound ID	27 (3.1)	885 (39.9)	1331 (60.1)
Academic level			
Individuals ever had difficulties in academic performance	735 (58.6)	1254 (56.6)	962 (43.4)
Individuals assessed for academic difficulties	359 (48.8)^b^	NA	NA
Neuropsychological level			
Individuals ever had any neuropsychological skill assessed	564 (41.6)	1355 (61.1)	861 (38.9)
Individuals with performance <5th percentile	314 (55.7)^c^	NA	NA

*ASD* autism spectrum disorder, *ADHD* attention deficit hyperactivity disorder, *ID* intellectual disability, *IQ* intelligence quotient, *NA* not applicable, *TAND* TSC-associated neuropsychiatric disorders

*Missing data includes unknown option ticked for TAND features in eCRF by patient/investigator

^a^Percentages calculated considering only available data and excluding missing data unless otherwise specified

^b^Percentages calculated based on the number of individuals with reported academic difficulties as denominator

^c^Percentages calculated based on the number of individuals who had neuropsychological skill assessed as denominator

#### Behavioural level

The most common behavioural problems (reported in > 10% of participants) were overactivity (45%), sleep difficulties (43.9%), impulsivity (42.7%), anxiety (33.3%), mood swings (29.8%), severe aggression (24.3%), depressed mood (19.2%), self-injury (15.5%), and obsessions (14%).

#### Psychiatric level

ASD was reported in 21.1% (314/1486), ADHD in 19.1% (268/1404), anxiety disorder in 9.7% (133/1365), depressive disorder in 6.1% (84/1371), and “other” psychiatric disorders were reported in 8.4% (115/1377) of participants. The median age at diagnosis of neuropsychiatric disorders were 5 years for ASD (mean, 7.8 years; range, < 1–40), 6 years for ADHD (mean, 7.8 years; range, < 1–38), 13.5 for anxiety disorder (mean, 17.4 years; range, < 1–50), 21 years for depressive disorder (mean, 24.3 years; range, 3–49), and 11 years for “other” psychiatric disorders (mean, 14.1 years; range, < 1–59).

#### Intellectual level

An IQ assessment was available for 885 participants (39.9%). Of these, 393 participants (44.4%) had normal intellectual ability, while mild, moderate, severe, and profound degrees of ID were observed in 28.1% (249/885), 15.1% (134/885), 9.3% (82/885), and 3.1% (27/885), respectively.

#### Academic and neuropsychological levels

Academic/scholastic difficulties, classified as learning disorders in DSM-5, such as mathematics, reading, writing, or spelling, were noted in 58.6% (735/1254) of participants. Neuropsychological skills were formally assessed in 41.6% (564/1355) of participants, and neuropsychological deficits (performance <5th percentile) were identified in 55.7% (314/564) of those evaluated.

### TAND features in children vs adults

Some differences were observed in the frequencies of TAND features between children (aged ≤18 years), and adults (aged > 18 years) (Table 2). At the behavioural level, the rates of overactivity and impulsivity were significantly higher for children than adults (54.8% vs 21.4% and 46.7% vs 33.2% respectively, *p* < 0.001), while rates of anxiety, mood swings, depressed mood, obsessions, psychosis and hallucinations were significantly higher in adults than children (50.9% vs 25.8%; 40.8% vs 25.2%; 43.9% vs 8.2%; 19.2% vs 11.8%; 11.3% vs 3%; 10.3% vs 0.6%, respectively, *P* < 0.001 for all except obsessions, *P* < 0.01). Interestingly, some behavioural manifestations showed similar rates in children and adults. For instance, sleep difficulties and severe aggression were very similar between children and adults (Table 2). At the psychiatric level, ASD and ADHD were reported at higher rates in children than in adults (23.1% vs 16.1%; 22.4% vs 10.5%; *p* = 0.0029 and P < 0.001 respectively), while rates of anxiety disorder and depressive disorder were higher in adults than in children (16.8% vs 7%; 16.3% vs 2.1% respectively; *P* < 0.001). We observed no major differences in the rates of academic difficulties and neuropsychological skills between children and adults (Table 2). However, highly significant difference was observed between the rates of intellectual disability between children and adults (Table 2).

**Table 2:** TAND Features in Children vs Adults

TAND Features	Children (≤ 18 Years) With Manifestation, (*n* = 1410) n (%)	Adults (> 18 Years) With Manifestation, (*n* = 806) n (%)	*p* value
Behavioural level^a^			
Overactivity	290 (54.8)	47 (21.4)	< 0.0001
Sleep difficulties	225 (43.4)	106 (45.1)	0.6531
Impulsivity	244 (46.7)	73 (33.2)	0.0006
Anxiety	130 (25.8)	110 (50.9)	< 0.0001
Mood swings	128 (25.2)	86 (40.8)	< 0.0001
Severe aggression	121 (22.9)	62 (27.4)	0.1850
Depressed mood	41 (8.2)	98 (43.9)	< 0.0001
Self-injury	73 (13.9)	44 (19.2)	0.0625
Obsessions	59 (11.8)	41 (19.2)	0.0085
Psychosis	15 (3.0)	25 (11.3)	< 0.0001
Hallucinations	3 (0.6)	23 (10.3)	< 0.0001
Psychiatric level^a^			
ASD	246 (23.1)	68 (16.1)	0.0029
ADHD	227 (22.4)	41 (10.5)	< 0.0001
Anxiety disorder	68 (7)	65 (16.8)	< 0.0001
Depressive disorder	21 (2.1)	63 (16.3)	< 0.0001
Intellectual level^b^			
Normal IQ	283 (43.5)	110 (47.0)	
Mild ID	202 (31)	47 (20.1)	
Moderate ID	101 (15.5)	33 (14.1)	0.0005^e^
Severe ID	51 (7.8)	31 (13.2)	
Profound ID	14 (2.2)	13 (5.6)	
Academic level			
Individuals ever had difficulties in academic performance^a^	506 (60.5)	229 (54.8)	0.0517
Individuals assessed for academic difficulties^c^	273 (54.0)^c^	86 (37.6)^c^	0.0785
Neuropsychological level			
Individuals ever had any neuropsychological skill assessed^a^	414 (42.5)	150 (39.4)	0.2926
Individuals with performance <5th percentile^d^	235 (56.8)^d^	79 (52.7)^d^	0.5333

*ASD* autism spectrum disorder, *ADHD* attention deficit hyperactivity disorder, *ID* intellectual disability, *IQ* intelligence quotient, *TAND* TSC-associated neuropsychiatric disorders

^a^Percentages calculated considering only available data and excluding missing data unless otherwise specified

^b^Percentages calculated based on the number of individuals with IQ assessment available in each age group: ≤ 18 years, *n* = 585; > 18, *n* = 234)

^c^Percentages calculated based on the number of individuals with reported academic difficulties as denominator

^d^Percentages calculated based on the number of individuals who had neuropsychological skill assessed as denominator

^e^Chi-square test showing association between children and adults across all levels of IQ

### TAND features by age bands

Frequencies of all the TAND features by age bands at consent are depicted in Table 3. The majority of TAND behavioural characteristics showed varying frequencies across age bands, but anxiety and depressed mood showed a clear pattern of increased frequency across increasing age bands. Interestingly, the rates of diagnoses of anxiety and depressive disorder showed a pattern that was not entirely consistent with the behavioural observations for anxiety and depressed mood. Severe and profound ID was reported at low rates in the youngest age group (3%), increasing in the under 10-year olds (6.6 and 9.7%), and ranging between 12.7–19.3% in the older age bands.

**Table 3:** TAND Features According to Age Bands (Based on Age at Consent)

TAND Features	Age at Registry Consent, Years						
	≤ 2	> 2to ≤ 5	> 5 to ≤ 9	> 9 to ≤ 14	> 14 to ≤ 18	> 18 to ≤ 40	> 40
	*n* = 283	*n* = 301	*n* = 335	*n* = 307	*n* = 184	*n* = 579	*n* = 227
Behavioural level^a^							
Overactivity	21 (53.8)	77 (65.3)	94 (60.6)	67 (47.5)	31 (40.8)	41 (23.7)	6 (12.8)
Sleep difficulties	24 (64.9)	43 (38.4)	61 (40.7)	68 (48.6)	29 (36.3)	70 (38.5)	36 (67.9)
Impulsivity	12 (31.6)	54 (47.4)	79 (52.0)	70 (49.3)	29 (38.2)	67 (38.3)	6 (13.3)
Anxiety	2 (5.6)	24 (21.2)	33 (22.9)	45 (33.3)	26 (34.2)	74 (44.6)	36 (72)
Mood swings	5 (13.5)	18 (16.1)	39 (25.8)	36 (27.1)	30 (40.5)	77 (46.4)	9 (20)
Severe aggression	2 (5.4)	15 (13.0)	45 (29.2)	40 (27.8)	19 (24.4)	57 (31.7)	5 (10.9)
Depression mood	0	0	10 (6.9)	17 (12.7)	14 (18.7)	68 (40.2)	30 (55.6)
Self-injury	3 (7.9)	9 (7.8)	28 (18.7)	23 (16.1)	10 (12.7)	37 (20.6)	7 (14.3)
Obsessions	2 (5.6)	7 (6.3)	15 (10.3)	22 (16.3)	13 (17.6)	33 (19.9)	8 (17.0)
Psychosis	0	2 (1.8)	4 (2.8)	7 (5.2)	2 (2.6)	22 (12.6)	3 (6.3)
Hallucinations	0	0	0	3 (2.3)	0	21 (11.9)	2 (4.3)
Psychiatric level^a^							
ASD	18 (10.8)	49 (22.9)	73 (26.2)	68 (26.3)	38 (26)	65 (18.6)	3 (4.2)
ADHD	11 (7.1)	50 (24.2)	82 (31.1)	61 (24.5)	23 (16.7)	39 (12.1)	2 (2.9)
Anxiety disorder	1 (0.6)	8 (3.9)	15 (5.9)	31 (13.1)	13 (10.4)	51 (16.1)	14 (19.7)
Depressive disorder	0	3 (1.4)	2 (0.8)	8 (3.4)	8 (6.3)	44 (14.2)	19 (25)
Intellectual level^b^							
Normal IQ	37 (56.1)	72 (52.9)	74 (39.8)	64 (37.9)	36 (38.3)	82 (42.7)	28 (66.7)
Mild ID	18 (27.3)	38 (27.9)	58 (31.2)	56 (33.1)	32 (34.0)	43 (22.4)	4 (9.5)
Moderate ID	9 (13.6)	17 (12.5)	36 (19.4)	25 (14.8)	14 (14.9)	30 (15.6)	3 (7.1)
Severe ID	1 (1.5)	9 (6.6)	13 (7.0)	18 (10.7)	10 (10.6)	27 (14.1)	4 (9.5)
Profound ID	1 (1.5)	0	5 (2.7)	6 (3.6)	2 (2.1)	10 (5.2)	3 (7.1)
Academic level							
Individuals ever had difficulties in academic performance^a^	1 (1.3)	48 (36.9)	167 (69.3)	193 (75.7)	97 (73.5)	195 (59.6)	34 (37.4)
Individuals assessed for academic difficulties^c^	0	20 (41.7)	93 (55.7)	103 (53.4)	57 (58.8)	77 (39.5)	9 (26.5)
Neuropsychological level							
Individuals ever had any neuropsychological skill assessed^a^	31 (20.4)	69 (32.4)	121 (48.2)	124 (53.2)	69 (55.2)	129 (42.7)	21 (26.6)
Individuals with performance <5th percentile^d^	12 (38.7)	35 (50.7)	69 (57.0)	78 (62.9)	41 (59.4)	72 (55.8)	7 (33.3)

*ASD* autism spectrum disorder, *ADHD* attention deficit hyperactivity disorder, *IQ* intelligence quotient, *ID* intellectual disability, *TAND* TSC-associated neuropsychiatric disorders. Values are expressed as n (%)

^a^Percentages calculated considering only available data and excluding missing data

^b^Percentages calculated considering individuals with IQ assessment available in each age group: ≤ 2 years (*n* = 66); > 2 to ≤5 years (*n* = 136); > 5 to ≤9 (*n* = 186); > 9 to ≤14 (*n* = 169); > 14 to ≤18 (*n* = 94); > 18 to ≤40 (*n* = 192); > 40 (*n* = 42)

^c^Percentages calculated based on the number of individuals with reported academic difficulties as denominator

^d^Percentages calculated based on the number of individuals who had neuropsychological skill assessed as denominator

### TAND and genotype

Molecular testing for genetic mutations was performed in 1000 participants (45.1%). Of them, 197 had *TSC1* mutations, 644 had *TSC2* mutations, and 144 had no mutation identified (NMI). At the behavioural level, *TSC2* mutations were associated only with significantly higher frequency than *TSC1* for self-injury (15.8% vs 6.3%, *P* = 0.0288; Table 4). At the psychiatric level, ASD was observed at significantly higher frequency in participants with *TSC2* than those with *TSC1* mutations (28.6% vs 12.2%, *P* < 0.001). ADHD, anxiety disorder and depressive disorder were not significantly different between the two genotypes, but it was interesting to observe that all three showed higher absolute frequencies in association with *TSC1* rather than *TSC2* (ADHD *TSC1 = 17.6%; TSC2* = 16%, *P* = 0.6881; Anxiety disorder *TSC1 = 10.1%; TSC2 =* 8.6%; *P* = 0.7809; Depressive disorders *TSC1 = 10%; TSC2 = 5.2%*; *P* = 0.0509) (Table 4). The frequencies in behavioural and psychiatric manifestations of individuals with NMI ranged sometimes between and sometimes higher or lower than *TSC1* and T*SC2* (Table 4).

**Table 4:** TAND Features by Genotype^*^

TAND Features	Individuals With *TSC1* Mutation (*n* = 197) n (%)^a^	Individuals With *TSC2 *Mutation, (*n* = 644) n (%)^a^	Individuals With no TSC Mutation Identified (NMI), (*n* = 144) n (%)^a^	Statistical difference between *TSC1* and *TSC2* *p* Value^e^
Behavioural level				
Overactivity	27 (40.9)	112 (43.4)	24 (45.3)	0.8788
Sleep difficulties	31 (48.4)	129 (50.6)	30 (54.5)	0.5656
Impulsivity	32 (49.2)	107 (41.6)	23 (45.1)	0.1773
Anxiety	24 (38.7)	82 (33.6)	17 (34.0)	0.5519
Mood swings	14 (21.5)	64 (26.6)	15 (29.4)	0.4907
Severe aggression	14 (21.2)	56 (21.5)	11 (20.8)	0.9290
Depression mood	15 (24.2)	46 (18.7)	12 (23.5)	0.2651
Self-injury	4 (6.3)	41 (15.8)	8 (14.5)	0.0288
Obsessions	8 (13.1)	35 (14.2)	8 (16.0)	0.9140
Hallucinations	3 (4.8)	7 (2.8)	2 (3.9)	0.3960
Psychosis	4 (6.5)	9 (3.6)	3 (5.8)	0.2865
Psychiatric level				
ASD	18 (12.2)	138 (28.6)	17 (17.5)	< 0.0001
ADHD	24 (17.6)	72 (16.0)	14 (14.9)	0.6881
Anxiety disorder	14 (10.1)	38 (8.6)	8 (9.2)	0.7809
Depressive disorder	14 (10.0)	23 (5.2)	7 (8.0)	0.0509
Intellectual level^b^				
Normal IQ	62 (66.7)	123 (42.0)	41 (64.1)	
Mild ID	15 (16.1)	75 (25.6)	14 (21.9)	
Moderate ID	11 (11.8)	57 (19.5)	3 (4.7)	0.001^f^
Severe ID	5 (5.4)	30 (10.2)	6 (9.4)	
Profound ID	0	8 (2.7)	0	
Academic level				
Individuals ever had difficulties in academic performance	63 (49.2)	240 (63.5)	53 (58.2)	0.0051
Individuals assessed for academic difficulties	34 (54.0)^c^	132 (55.0)^c^	25 (47.2)^c^	0.5391
Neuropsychological level				
Individuals ever had any neuropsychological skill assessed	67 (51.1)	216 (49.4)	46 (51.1)	0.7697
Individuals with performance <5th percentile	26 (38.8)^d^	136 (63.0)^d^	19 (41.3)^d^	0.0024

*ASD* autism spectrum disorder, *ADHD* attention deficit hyperactivity disorder, *ID* intellectual disability, *IQ* intelligence quotient, *TAND* TSC-associated neuropsychiatric disorders

^*^Molecular testing for genetic mutations was performed in 1000 (45.1%) individuals. Of these, 644 (64.4%) had a *TSC2* gene mutation, 197 (19.7%) had a *TSC1* gene mutation, 6 had both *TSC1* and *TSC2* gene mutations and 144 (14.4%) had no mutations. Data were not available for 9 individuals

^a^Percentages calculated considering only available data and excluding missing data unless otherwise specified

^b^Percentages calculated considering individuals with IQ assessment available in each group: Individuals with *TSC1* mutation, *n* = 93; individuals with *TSC2* mutation, *n* = 293)

^c^Percentages calculated based on the number of individuals with reported academic difficulties as denominator

^d^Percentages calculated based on the number of individuals who had neuropsychological skill assessed as denominator

^e^*TSC1* vs *TSC2*

^f^Chi-square test showing association between children and adults across all levels of IQ

Of the 93 participants with *TSC1* mutation who had been evaluated using IQ-type tests, 62 (66.7%) had normal intellectual ability, 15 (16.1%) had mild ID, 11 (11.8%) had moderate ID, 5 (5.4%) had severe ID, and no participant had profound ID. Among 293 participants with *TSC2* mutation who had been evaluated using IQ-type tests, 123 (42%) had normal intellectual ability, 75 (25.6%) had mild ID, 57 (19.5%) had moderate ID, 30 (10.2%) had severe ID, and 8 (2.7%) had profound ID. Significant difference was observed between *TSC1* and *TSC2* groups for IQ levels/categories. (*P* = 0.001, Table 4).

Academic/scholastic difficulties were more common in individuals with *TSC2* mutation than those with *TSC1* mutation (63.5% vs 49.2%; *P* = 0.0051, Table 4). More individuals with *TSC2* mutation had neuropsychological performance scores falling below the 5th percentile compared to those with *TSC1* mutation (63% vs 38.8%, *P* = 0.0024). Individuals with NMI showed IQ, academic and neuropsychological profiles in between the frequencies of the group with *TSC1* and *TSC2*.

## Discussion

The TOSCA registry allowed exploration of the frequency of a wide range of TAND features in the largest cohort of TSC reported to date. We set out to examine the overall TAND profile, to compare childhood and adult patterns, age-based patterns, and genotype-TAND correlations. Results showed lower rates of behavioural and psychiatric disorders than previously reported, but similar rates of ID [3, 6, 8, 12]. The very high rates of non-reported or missing data in this study (in excess of 60% for behavioural and intellectual levels) may, at least in part, have contributed to the lower rates observed. The rates of academic difficulties and neuropsychological deficits, reported in this study for the first time, were very high and suggested that between half and two-thirds of individuals with TSC will have difficulties in these two TAND levels. In spite of relatively similar rates of ID between children and adults, we observed a pattern of higher overactivity and impulsivity in children, and higher rates of anxiety and depressed mood in adults. Interestingly, some TAND characteristics such as sleep problems and severe aggression remained high across all age bands, suggesting persistence of these difficulties across the lifespan. With regards to genotype-TAND correlations, we observed a genotype-intellectual phenotype correlation and a higher frequency of ASD in association with *TSC2,* similar to previous reports [8, 26]. Interestingly, fewer other TAND-genotype correlations were observed. Self-injury, ASD, academic difficulties and neuropsychological deficits were the only other significant correlations with *TSC2*. Given that all of these are known to be strongly correlated with intellectual level [12], and given the differences observed here in IQ groups, these results have to be treated with caution and should be explored in relation to matched or stratified IQ groups in future studies. We also observed a potential pattern of more depressed and anxious mood, and higher rates of anxiety and depressive disorders in association with *TSC1* mutations. This was a novel observation not previously reported in the literature, but the same caveat in relation to IQ as potential confounder requires exploration in future studies. The TAND profile of individuals with NMI was also a novel finding. Intellectual, academic and neuropsychological profiles seemed to fall between the frequencies of those with *TSC1* and *TSC2* mutations, mapping onto intellectual findings previously reported [8, 26]. However, the same pattern was not seen in behavioural and psychiatric manifestations.

About 7% of individuals in the general population are expected to have clinically significant behavioural problems [33]. However, much higher rates of behavioural difficulties are reported in patients with TSC [7]. In a pilot validation study of the TAND checklist, all the participants (*n* = 62) had reported at least one lifetime TAND behavioural difficulty, 97% had ≥2 behavioural difficulties and 89% reported ≥6 behavioural difficulties [6]. The findings in this large-scale international study confirm the high rates of a wide range of behavioural problems in TSC. The comparison between children and adults showed a pattern of lower overactive/impulsive behaviours in adults but higher anxiety or depressed mood. These findings map well onto typical age-based expectations in psychopathology [8, 9, 12]. However, our results also highlighted the fact that behavioural difficulties occurred across all ages in individuals with TSC. These findings underline the importance of expecting and evaluating for a changing profile of TAND difficulties from childhood into adulthood, as recommended in TAND assessment guidelines [2, 34, 35].

Among the psychiatric problems associated with TSC, ASD, and ADHD are the most common neurodevelopmental disorders in children, and anxiety/mood disorders the most common in adults [8]. The variable rates of ASD (17–68%) and ADHD (30–60%) reported in earlier studies can be understood based on different study methodologies, diagnostic criteria used, and level of intellectual ability of participants [7, 8, 36]. In this study, ASD and ADHD were reported in 21.1% and 19.1% of participants, respectively. As highlighted in our baseline paper [27] the diagnosis of ASD, in particular, was made very late (mean age, 7.8 years; median age, 5 years; range, 0–38 years) [28]. In spite of very high rates of anxiety and depressed mood symptoms in the cohort, the rates of diagnoses of anxiety disorders or depressive disorders were surprisingly low in our study (anxiety disorder, 9.7%; depressive disorder, 6.1%). Taking together the high rates of non-reported or missing data, late ages of diagnoses and frequencies observed in our cohort, we suggest that psychiatric disorders are underdiagnosed and potentially diagnosed late in individuals with TSC. The low rates of ASD and ADHD observed in adults here, may suggest a cohort effect where adults were not assessed for developmental disorders in the past decades.

In line with previous reports, [8, 26, 37] the TOSCA registry showed a genotype-intellectual phenotype pattern suggesting a greater likelihood of ID in participants with *TSC2* than those with *TSC1* mutations. However, it was also important to note that only 66.7% of those with *TSC1* had normal intellectual ability, suggesting that a third of individuals with *TSC1* may have ID. Similarly, even though *TSC2* mutations were more likely to be associated with ID, 42% of all individuals with *TSC2* mutations had normal intellectual ability. Our findings therefore reinforce the message that genotype (*TSC1* vs *TSC2*) is not a clinically helpful predictor of intellectual ability at an individual level [26].

The differences between those with *TSC1* and *TSC2* mutations observed in other aspects of TAND were of interest, particularly as all previous genotype-phenotype studies have suggested a more “severe” phenotype associated with *TSC2* [38]. The possibility that specific aspects of TAND may be more likely in association with *TSC1* is therefore a potentially important observation. We acknowledge that a *TSC1* vs *TSC2* differentiation may be highly oversimplistic, given that specific TSC mutations may be associated with very different functional consequences at a biochemical level [16, 26].

The challenge around unreported and missing TAND data in this study was significant. Fewer than 40% of behavioural data were available and only 39.9% of participants in the TOSCA registry received an evaluation of their intellectual ability. The overall rates of ID were consistent with previous studies, [8, 12] but we did not observe a bimodal distribution of intellectual ability as previously reported [23, 25, 39, 40]. Given that only about half of the TOSCA cohort received a formal evaluation for IQ, it is possible that more severely impaired individuals may not have been referred for an assessment of their intellectual or developmental quotient (as was done in the population-based study that observed the clear bimodal pattern) [39]. Academic data were not reported or were missing in 43.4% of the cohort and 38.9% were not reported or were missing regarding neuropsychological skills. Only 48.8% of those with academic difficulties ever received evaluations, and only 41.6% were evaluated for neuropsychological deficits. Whilst the very high rates of academic and neuropsychological deficits in TSC were a novel finding in the TOSCA registry and underlines the recommendation that all children with TSC should receive evaluation of their academic and neuropsychological needs, [2, 35] the very low proportion assessed raises significant clinical concern.

## Conclusions

Together, findings from the TOSCA registry emphasize the magnitude of neuropsychiatric disorders in TSC, which has an enormous impact on quality of life of individuals with TSC and their families. However, there was a high proportion of non-reported or missing data, which may have impacted the overall findings of the study. Due to the observational nature of the registry, only data already available from clinical practice were collected. Moreover, considering the disease complexity and the fact that individuals with TSC were not always followed for all disease manifestations by the site involved in the registry, ensuring report of all disease manifestations of each participant was a major challenge. We therefore acknowledge the potential ascertainment biases that may come with large-scale clinic-based natural history studies such as this one.

There is clearly a highly dynamic interaction between development, genotype, intellectual ability, epilepsy, anti-epilepsy and other pharmacological treatments, and environment in TSC. Future studies should therefore aim to examine these interrelations in an integrated way using suitable multilevel computational modelling. We also acknowledge that further evaluations will be required to examine TAND in relation to intellectual level and sex. In addition, efforts are underway to determine if there may be “natural TAND clusters” consisting of natural groupings of TAND characteristics within and between individuals with TSC across levels of TAND [7, 13]. The TOSCA cohort may provide a powerful dataset to examine this possibility further.

In spite of the limitations inherent to a large-scale natural history study, results provide strong data to encourage clinicians to evaluate for neuropsychiatric comorbidity in all children and adults with TSC. The 2012 TSC surveillance and management guidelines recommend annual screening for TAND [34]. A simple tool called the TAND Checklist has been developed and validated in a pilot study that could provide easy guidance to healthcare professionals in assessing neuropsychiatric difficulties in each individual with TSC [3, 5, 6].

## Abbreviations

ADHD: Attention deficit hyperactivity disorder
ASD: Autism spectrum disorders
ID: Intellectual disability
IQ: Intelligence quotient
TAND: TSC-associated neuropsychiatric disorders
TOSCA: TuberOus SClerosis registry to increase disease Awareness
TSC: Tuberous sclerosis complex
